# Skin Diseases

**Published:** 1871-10

**Authors:** J. W. Southworth


### Skin Diseases

*The Students' Classification of Skin Diseases.* By J. W. SOUTHWORTH, M. D.

We have received from the author the following classification of Skin diseases, which we publish for the benefit of students and others interested.

CLASS ONE.

Inflammatodermæ which are Non-contagious, and chiefly Diathetical.

*Erythematous Group.*—Erythema, (Simplex), Urticaria, (Simplex), Roseola, Erysipelas, (Simplex), Pellagra, Acrodynia.

*Eczematous Group.*—Includes Impetigo, Lichen, Eczema, Pityriasis, ‡Psoriasis, Gutta Rosacea.

*Bullous Group.*—Herpes, (Simplex), Miliaria, (Simplex), Pemphigus, (Simplex.)

*Furunculous Group.*—Papulae, (Simplex), Pastulae, (Simplex), Tuberculæ, (Simplex), Furunculæ, Carbunculæ.

*Scrofulous Group.*—Scrofulodermæ.

*Lupous Group.*—Lupodermæ.

CLASS TWO.

Inflammatodermæ which are Contagious or Contagious and Infectious and depend on the presence of a zymotic (organic,) Poison in the Blood.

‡ The Alphos of Wilson.

*Septicemic Group.*—Septic Erythema, Septic Erysipelas, Malignant Pustule.

*Syphilitic Group.*—Syphilodermæ.

*Leprous Group.*—Leprodermæ.

CLASS THREE.

Inflammatodermæ which depend on the presence of Parasites.

*Fungoid, or Sporular Group*—Tinca Favosa from Achorian Schoenleinii, Tinca Circinata from Trichophyton Tonsurans, Tinca Versicolor from Microsporon Furfur.

*Animacular, or Verminous Group.*—Scabies from Acarus Scabei, Rouget from Acarus Autumnalis, Phtheirasis from Pediculus Corporis, Capitis, &c.

*Helminthic, or Vermicular Group.*—Malis Filaria, Malis Œstri,

CLASS FOUR.

Hemodyscrasic Affections.

Purpura Hemorrhagica.

CLASS FIVE.

Cancerous Affections.

Epithilial, Melanotic, Tubercular and Scirrhus Cancer.

CLASS SIX.

Developmental and Nutritive Affections.

*Trophic Group.*—Exoderma, Icthyosis, Sauriosis, Hypertrophy, Atrophy, Cacotrophia, Excrescences, Verrucæ, (Simplex), Clavus, Pilary Growths, Papilary Growths, Induration, Keloid, and Barbadoes Leg.

*Chromotogenous Group.*—Chloasma, Leucodermæ, Cyanodermæ, Xanthoderæ, Melanodermæ.

*Nevic Group.*—Venous Nevi, Arterial Nevi, Arterio-Venous Nevi, Pilary Nevi, Pigmento-Pilary Nevi, Hypertrophic Nevi.

CLASS SEVEN.

Neurotic Affections.

Neuralgia, Anesthesia, Hyperesthesia, Prurigo, Pruritus, Chloasma.

CLASS EIGHT.

Glandular Affections.

*Of the Sudoriferous System.*—Atrophy, Anidrosis, Ephidrosis, Osmidrosis, Hemidrosis, Chromidrosis, Hyperemia, Hydro-Adenitis.

*Of the Sebiperous System.*—Acne, Atrophy, Seborrhœa, Comedones, Sebaceous Tumor, Sebaceous Tubercles, Horny Growths, Adenitis.

CLASS NINE.

Traumatic Affections.

Burns, Scalds, Frost Bite, Chilblain.

CLASS TEN.

Chief Affections of the Appendages.

Simple, Syphilitic and Parasitic Alopecia, Hirsuties, or Excess of Hair, Atrophy of Hair, &c., Trichiasis, or Change in Direction, Tinea Favosa, or Purulent Parasitic Folliculitis, Tinea Kerion, or Pustular Parasitic Folliculitis, Tinea Circinata Capitis, or Ringworm of Scalp, Tinea Sycosis Menti, or Ringworm of Beard, Sycosis Mentagra, or Non-Parasitic Sycosis, Hordeolum, or Pustular Folliculitis of Eyelashes.

Simple, Traumatic, Scrofulous and Syphilitic Onychia, Degeneration, Incurvation and Alopecia Ungualis.

*Exanthematous Group.*—Variola, Vaccinia, Varicella, Varioloid, Rubeola, Scarlatina, † Erysipelas.

† Endemic and Epidemic forms.

